# P-967. Antimicrobial Stewardship Program Supported by Infectious Disease Specialists and Consultations at a University-Affiliated Hospital Under Multiple Antimicrobial Shortages: Bayesian Structural Time Series Analysis

**DOI:** 10.1093/ofid/ofaf695.1167

**Published:** 2026-01-11

**Authors:** Naoya Itoh, Nobumasa Okumura, Shunsuke Kuriki, Chiharu Wachino, Takanori Kawabata, Nana Akazawa-Kai

**Affiliations:** Nagoya City University, Nagoya, Aichi, Japan; Nagoya City University, Nagoya, Aichi, Japan; Nagoya City University, Nagoya, Aichi, Japan; Nagoya City University, Nagoya, Aichi, Japan; National Cerebral and Cardiovascular Center, Suita, Osaka, Japan; Nagoya City University, Nagoya, Aichi, Japan

## Abstract

**Background:**

Antimicrobial shortages have become increasingly common owing to manufacturing issues and sudden surges in demand. Thus, individualized antimicrobial optimization supported by infectious disease (ID) specialists is essential under such constraints. In this study, we evaluated the effects of a 12-month antimicrobial stewardship program (ASP) supported by ID specialists and consultations at a university-affiliated hospital in Japan during the ongoing antimicrobial shortages.

**Figure 1.**
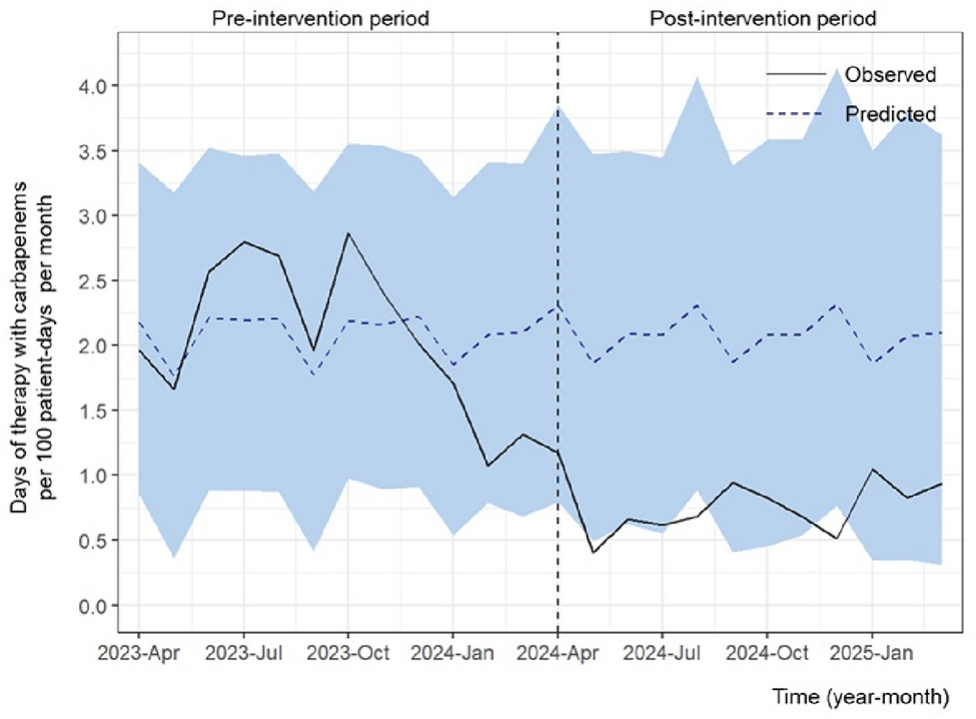
The days of therapy with carbapenems per 100 patient-days per month The days of therapy (DOT) with three carbapenems—imipenem/cilastatin, meropenem, and doripenem—per 100 patient-days per month is shown. The study period was divided into two phases: the pre-intervention period (April 1, 2023, to March 31, 2024) and the post-intervention period (April 1, 2024, to March 31, 2025). The light blue shaded area denotes the 95% credible interval around the predicted values. A significant reduction in DOT for the three carbapenems was observed (average effect: -1.31; 95% credible interval: -1.77 to -0.84).

**Figure 2.**
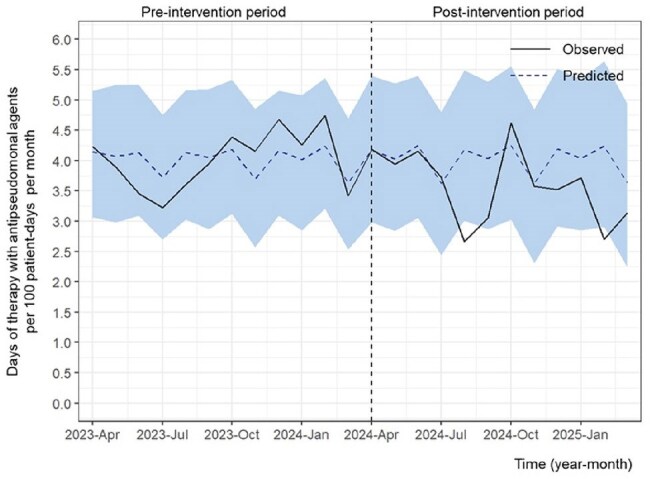
The days of therapy with antipseudomonal agents per 100 patient-days per month The days of therapy (DOT) with three antipseudomonal agents — cefepime, piperacillin/tazobactam, and ceftolozane/tazobactam—per 100 patient-days per month is shown. The study period was divided into two phases: the pre-intervention period (April 1, 2023, to March 31, 2024) and the post-intervention period (April 1, 2024, to March 31, 2025). The light blue shaded area denotes the 95% credible interval around the predicted values. The DOT significantly decreased for three antipseudomonal agents (average effect: -0.44; 95% credible interval, -0.83 to -0.05).

**Methods:**

We conducted a single-center retrospective observational study from April 1, 2023, to March 31, 2025, comparing pre-intervention (ASP without ID physicians) and post-intervention (ASP with ID physicians and a formal consultation service) periods. A Bayesian structural time series analysis adjusted for seasonality was used to assess the effects of the intervention.

**Figure:**
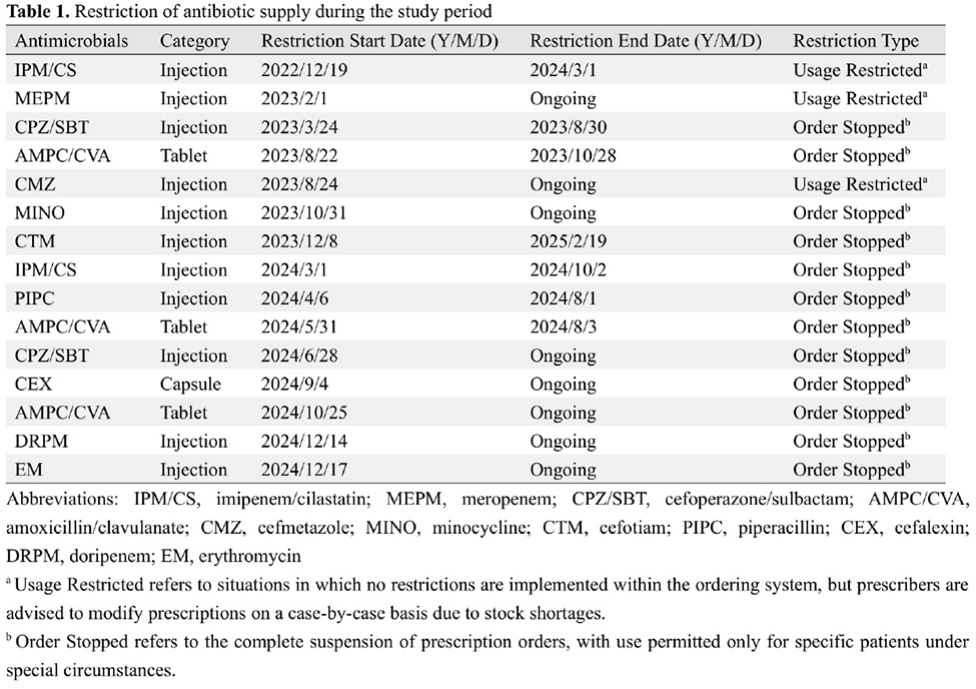

**Figure:**
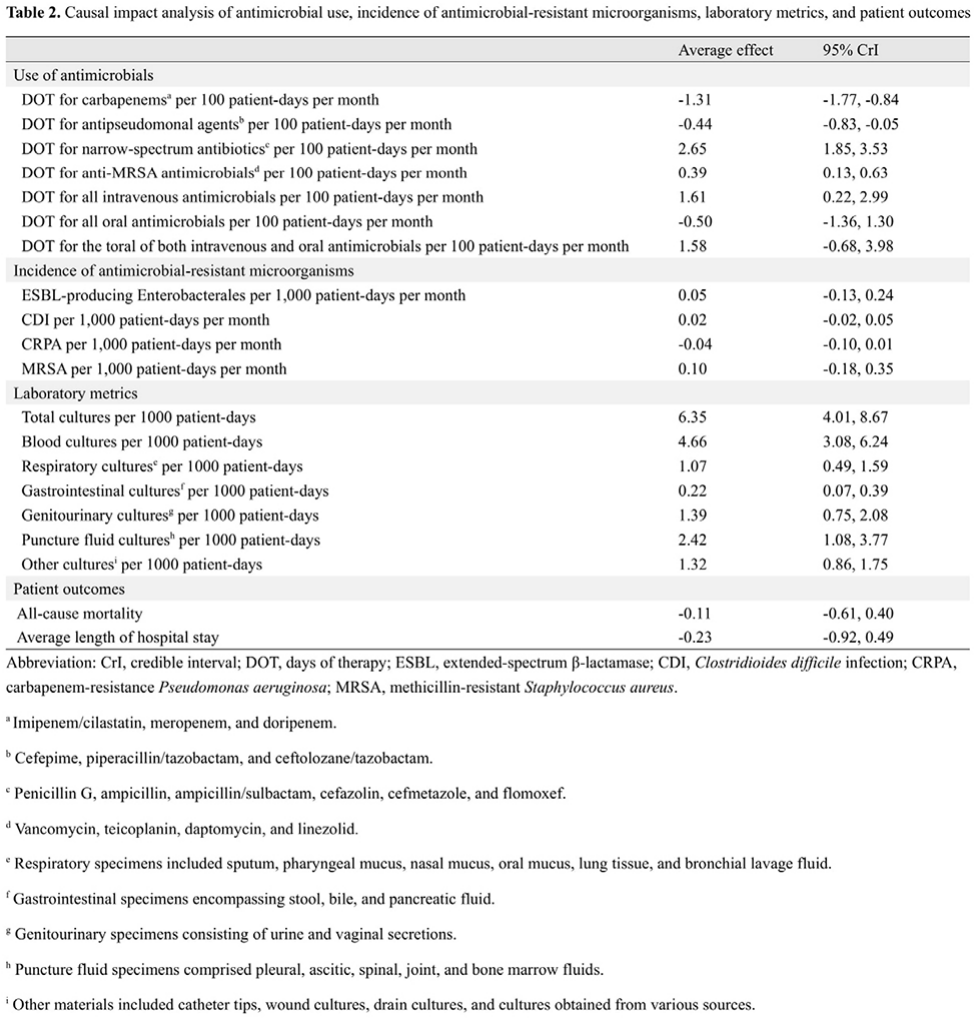

**Results:**

During the study period, 24,601 inpatients were included (11,782 before and 12,819 after the intervention). Shortages affected nine intravenous and two oral antibiotics (Table 1). The ASP team provided 1,110 feedback instances with a 74.1% acceptance rate, and 172 ID consultations were conducted during the post-intervention period. The days of therapy (DOT) significantly decreased for carbapenems (CARs) and antipseudomonal agents (Figures 1 and 2), whereas it increased for narrow-spectrum antibiotics, anti-methicillin-resistant *Staphylococcus aureus* antimicrobials, and all intravenous antimicrobials (Table 2). No significant change was observed in the total DOT for combined intravenous and oral agents. The number of inpatient specimens significantly increased; however, hospital-acquired resistant organisms, in-hospital mortality, and the length of hospital stay remained unchanged. The CARs purchase cost per patient-day significantly decreased, while the total antimicrobial costs per patient-day did not change significantly.

**Conclusion:**

An ASP supported by ID specialists and consultants significantly reduced CARs and antipseudomonal agent use without negatively affecting patient outcomes. These findings highlight the critical role of ID specialists in developing effective ASPs, particularly during periods of limited antimicrobial supply and increased clinical complexity.

**Disclosures:**

Naoya Itoh, MD, DTM&H, PhD, Asahi Kasei Pharma Corporation: Honoraria|AstraZeneca K.K: Honoraria|BD Co, Ltd: Honoraria|bioMérieux Japan Ltd: Honoraria|Gilead Sciences Inc: Honoraria|GlaxoSmithKline: Honoraria|Meiji Seika Pharma Co, Ltd: Honoraria|MSD K.K: Honoraria|Pfizer;: Honoraria|shimadzu co ltd: Grant/Research Support|Shionogi Co, Ltd: Honoraria

